# LZP is required for hepatic triacylglycerol transportation through maintaining apolipoprotein B stability

**DOI:** 10.1371/journal.pgen.1009357

**Published:** 2021-02-16

**Authors:** Jiao-Xiang Wu, Kun-Yan He, Zhuang-Zhuang Zhang, Yu-Lan Qu, Xian-Bin Su, Yi Shi, Na Wang, Lan Wang, Ze-Guang Han

**Affiliations:** 1 Key Laboratory of Systems Biomedicine (Ministry of Education) and Collaborative Innovation Center of Systems Biomedicine of Rui-Jin Hospital, Hongqiao International Institute of Medicine, Tongren Hospital, Shanghai Jiao Tong University School of Medicine, Shanghai, China; 2 Key Laboratory of Systems Biomedicine (Ministry of Education), Shanghai Center for Systems Biomedicine, Shanghai Jiao Tong University, Shanghai, China; 3 Shanghai-MOST Key Laboratory for Disease and Health Genomics, Chinese National Human Genome Center at Shanghai, Shanghai, China; University of Michigan Medical School, UNITED STATES

## Abstract

The conserved zona pellucida (ZP) domain is found in hundreds of extracellular proteins that are expressed in various organs and play a variety of roles as structural components, receptors and tumor suppressors. A liver-specific zona pellucida domain-containing protein (LZP), also named OIT3, has been shown to be mainly expressed in human and mouse hepatocytes; however, the physiological function of LZP in the liver remains unclear. Here, we show that *Lzp* deletion inhibited very low-density lipoprotein (VLDL) secretion, leading to hepatic TG accumulation and lower serum TG levels in mice. The apolipoprotein B (apoB) levels were significantly decreased in the liver, serum, and VLDL particles of LZP*-*deficient mice. In the presence of LZP, which is localized to the endoplasmic reticulum (ER) and Golgi apparatus, the ER-associated degradation (ERAD) of apoB was attenuated; in contrast, in the absence of LZP, apoB was ubiquitinated by AMFR, a known E3 ubiquitin ligase specific for apoB, and was subsequently degraded, leading to lower hepatic apoB levels and inhibited VLDL secretion. Interestingly, hepatic LZP levels were elevated in mice challenged with a high-fat diet and humans with simple hepatic steatosis, suggesting that LZP contributes to the physiological regulation of hepatic TG homeostasis. In general, our data establish an essential role for LZP in hepatic TG transportation and VLDL secretion by preventing the AMFR-mediated ubiquitination and degradation of apoB and therefore provide insight into the molecular function of LZP in hepatic lipid metabolism.

## Author summary

The metabolic diseases, such as obesity, atherosclerosis, and nonalcoholic fatty liver disease (NAFLD), are often characterized by increased triacylglycerol (TG) and cholesterol levels. TG is synthesized mainly in the liver, incorporated into Very Low-Density Lipoproteins (VLDLs), and then secreted into blood. During TG metabolism, apolipoprotein B (apoB) is necessary for the assembly and secretion of TG-rich VLDLs because it functions as the major component of VLDLs. In the present work, using a genetically engineered mouse model, we discovered that the liver-specific zona pellucida domain-containing protein (LZP)-deficient mice exhibit hepatic TG accumulation and low blood TG, which are eventually ascribed to the decrease of apoB protein. In physiological condition, the unnecessary apoB is usually ubiquitinated by E3 ligase AMFR, and subsequently degraded by ubiquitination proteasomes. Significantly, LZP can interact with apoB, and prevent the AMFR-mediated apoB degradation. Therefore, LZP deficiency destroys the protection for apoB, leading to the enhancement of apoB ubiquitination and degradation, and the secondary decrease of TG-rich VLDL secretion, which finally results in TG accumulation in liver and lower blood TG. This study reveals a novel regulator of TG-rich VLDL assembly and secretion, which could advance our knowledge on lipid metabolism.

## Introduction

The evolutionarily conserved zona pellucida (ZP) domain, consisting of ∼260 amino acid residues, is shared by hundreds of extracellular proteins expressed in a variety of epithelial cells. Previous studies have extensively demonstrated that ZP-domain proteins such as ZP1, ZP2 and ZP3, serving as structural components of egg coating, have physiological roles that are mainly restricted to reproduction [1,2]. In addition to their roles in animal fertilization, recent reports show that several ZP-domain proteins have impacts on some important biological functions, as mutations are linked to severe human diseases such as infertility, deafness, cancer, kidney and vascular diseases [3].

The ZP domain consists of a bipartite protein structural element comprising ZP-N and ZP-C regions and is relatively close to the C-terminus of the protein. The ZP domain is one of the most recognizable motifs in a polypeptide [4,5]. In addition, most ZP-domain proteins, which are usually glycosylated, possess an N-terminal signal peptide and either a C-terminal putative transmembrane domain (TMD) or a glycosyl phosphatidylinositol (GPI) anchor. The ZP domain plays a common role in protein polymerization, and thus, many ZP-domain proteins polymerize and assemble into long fibrils to form specialized extracellular matrices. However, some ZP-domain proteins, such as beta-glycan and endoglin, do not polymerize but serve as important membrane coreceptors for ligands derived from transforming growth factor-β (TGF-β) superfamily members [2]. ZP-domain proteins have been found in various tissues and organs and play a wide variety of roles, from structural components to receptors to tumor suppressors [4,5].

Interestingly, we previously isolated liver-specific zona pellucida domain-containing protein (LZP), also named OIT3, which is mainly expressed in human and mouse hepatocytes and is detectable in low amounts in kidney tubules [6]. The structure of the LZP molecule is evolutionarily conserved across multiple species, including mouse, rat and human, and includes a signal peptide, three EGF-like domains, one D8C domain, one consensus furin cleavage site (CFCS), and one ZP domain [6–8]. In rat and mouse kidney, LZP is anchored to the apical membrane of the thick ascending limb of the loop of Henle (TAL) cells and may interact with Tamm-Horsfall protein (THP), also a ZP-domain protein, in TAL cells and urine. Moreover, LZP may self-aggregate into spheres of different sizes [9]. LZP deficiency impaired uric acid reabsorption in renal tubules, and additional evidence indicated that LZP could maintain urate homeostasis by regulating the excretion and reabsorption of uric acid in renal tubules in cooperation with THP [10]. Although LZP, as a secreted protein, was decreased or even disappeared in 90% of the hepatocellular carcinoma specimens, overexpression or knockdown of LZP had no effect on cell proliferation, and no tumor was found in the livers of LZP-null mice as old as one and one-half years, which implied that LZP downregulation might have been a byproduct of hepatocarcinogenesis [7]. However, the real physiological function of LZP in the liver, where it is mainly expressed, remains unclear and should be further explored.

The liver plays a unique role in the regulation of nutrient metabolism, including glucose and lipid homeostasis. Dysregulation of hepatic functions is related to many common diseases. For example, some metabolic diseases, such as obesity, insulin resistance, atherosclerosis, and nonalcoholic fatty liver disease (NAFLD), are often characterized by increased triacylglycerol (TG) and cholesterol levels [11–13]. Hepatic TG metabolism consists of uptake fatty acids or *de novo* fatty acid synthesis from glucose, β-oxidation of the fatty acids, and storage in lipid droplets, where TG is further processed and then delivered to other tissues in the form of very low-density lipoprotein (VLDL) [14,15]. During TG metabolism, apolipoprotein B (apoB), a large (520-kDa) complex secretory protein, is necessary for the assembly and secretion of TG-rich VLDL particles because it functions as a main component in the particles.

Control over apoB secretion is achieved by cotranslational degradation of nascent apoB through the ubiquitin-proteasomal pathway, namely, endoplasmic reticulum (ER)-associated degradation (ERAD), which involves 70- and 90-kDa heat shock proteins, E3 ligase gp78 (AMFR) and multiple components of the proteasomal pathway. The degradation of apoB is known to be usually regulated through newly synthesized core lipids (triglyceride and cholesterol ester) and microsomal triglyceride transfer protein (MTTP) activity [16]. However, whether novel components also participate in the maintenance of apoB stability is unclear. Identification of novel components involved in the regulation of apoB degradation is expected to advance our understanding of the molecular mechanism of hepatic VLDL secretion.

LZP is highly expressed in the liver; however, the physiological function of LZP in the liver remains unclear. Importantly, in the present study, we demonstrate that LZP deficiency causes TG accumulation within hepatocytes, along with lower serum TG levels, by affecting VLDL secretion and apoB degradation. This is the first study, to our knowledge, to uncover the regulation of lipid homeostasis via a ZP-domain protein. The functional characterization of LZP in hepatic VLDL secretion will provide new insight into lipid metabolism.

## Results

### LZP-deficient mice exhibit lower serum TG levels and resistance to high-fat diet (HFD)-induced obesity

To date, the physiological functions of LZP in the liver, where it is highly expressed, have not been discovered. To explore LZP function, we first measured the different blood biochemistry indexes related to the liver function and found that the serum TG level was significantly lower in the *Lzp*^*-/-*^ mice fed a chow diet (CHD), which also had the reduced (but not significantly) low-density lipoprotein cholesterol (LDL-C). The other indexes showed no significant change (**S1 Fig and S1 Table**). In addition, no body weight or liver mass changes were observed in the *Lzp*^*-/-*^ mice fed a CHD.

**Fig 1:**
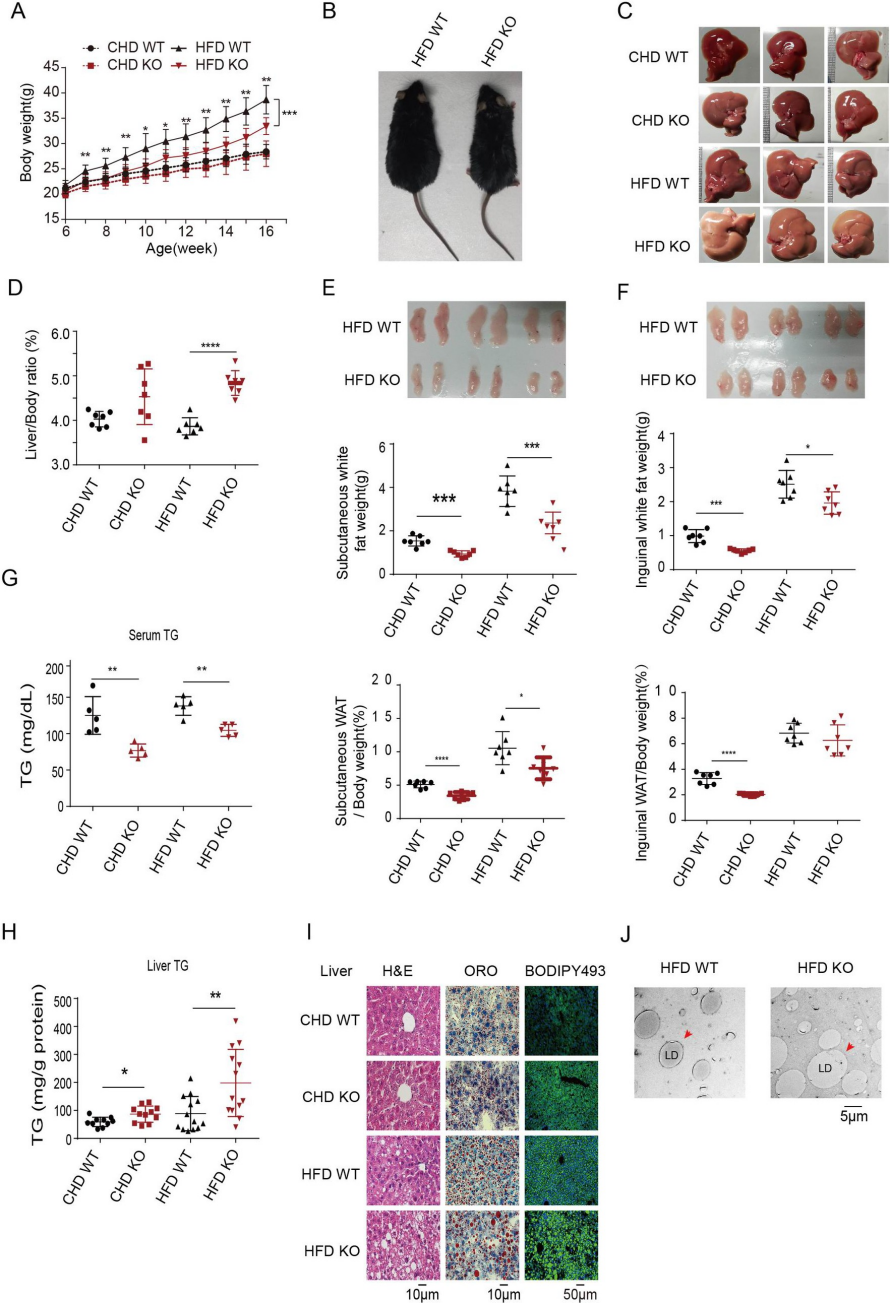
LZP-deficient mice exhibit lower serum triacylglycerol (TG). (A) Growth curve of wild type (WT) and *Lzp* knockout (KO) mice fed with chow diet (CHD) or high fat diet (HFD) for 10 weeks. Body weight was measured weekly (n = 7). (B, C) Representative whole body (B) and liver (C) images of WT and *Lzp* KO mice fed with CHD or HFD. (D) The liver/body ratios of WT and *Lzp* KO mice fed with CHD or HFD (n = 7). (E-F) The images, weights of subcutaneous white adipose tissue (gWAT) and inguinal WAT (iWAT) and, as well as their body ratios (n = 7). (G-H) Quantifications of serum and liver TG from 4-month-old mice that have been fed with CHD or HFD (n = 11–13). (I) Images of H&E staining (left panel, scale bar represents 10 μm), Oil Red O staining (middle panel, scale bar represents 10 μm), and fluorescence staining with Bodipy493 (right panel, scale bar represents 50 μm) on liver sections from 4-month-old WT and *Lzp* KO mice fed with CHD or HFD. (J) Transmission electron micrographs of liver sections from WT and *Lzp* KO mice fed with HFD. Scale bar represent 5μm. Data were expressed as means ± SEM, and statistically analyzed by student’s t-test or one-way ANOVA, *P < 0.05, **P < 0.01. Numerical values for each of the experiments represented are available in **S3 Table**.

Furthermore, we challenged *Lzp*^*-/-*^ mice with a high-fat diet (HFD) to assess the functions of LZP. Interestingly, despite of similar food intake (**S2A Fig and S2 Table**), the *Lzp*^*-/-*^ mice fed a HFD had lower body weight gains, smaller body sizes, larger livers and higher liver/body ratios than exhibited by their WT littermates (**Fig 1A–1D and S3 Table**). Moreover, both subcutaneous white adipose tissue (gWAT) and inguinal WAT (iWAT) fat pads were significantly smaller in the *Lzp*^*-/-*^ mice than they were in the WT littermates fed CHD or HFD. The respective gWAT/body and iWAT/body ratios further confirmed these phenotypes **(Fig 1E and 1F and S3 Table)**. The serum TG level, but not total cholesterol level, in the *Lzp*^*-/-*^ mice was significantly decreased, regardless of CHD or HFD challenge (**Figs 1G and S2B, left panel and S2 and S3 Tables**). However, the glucose and insulin tolerance tests did not show a significant difference between the *Lzp*^*-/-*^ and WT mice fed a CHD or HFD (**S2C Fig and S2 Table**), indicating that LZP loss did not affect glucose metabolism. In the presence or absence of LZP, insulin signaling, as indicated by the levels of phosphorylated insulin receptors and AKT, showed no obvious change in these isolated mouse hepatocytes (**S2D Fig**).

These collective data suggest that LZP is mainly involved in hepatic TG metabolism.

### LZP deficiency causes hepatic TG accumulation

To explore the mechanism by which TG level is reduced in the serum of *Lzp*^*-/-*^ mice, we examined the TG level in their livers, where TG is primarily synthesized and exported. The *Lzp*^*-/-*^ mice had significantly higher hepatic TG levels, but not total cholesterol levels, compared to those in the WT mice, and those fed a HFD showed greater increases in liver TG levels (**Figs 1H and S2B, right panel and S2 and S3 Tables**). Consistently, hematoxylin and eosin (H&E) staining also showed visible cytoplasmic fat vacuoles in the livers of the *Lzp*^*-/-*^ mice fed a CHD, which were even more noticeable in the livers of the *Lzp*^*-/-*^ mice fed a HFD (**Fig 1I, left panel)**. Staining with both oil red O and lipophilic fluorescent dye boron-dipyrromethene dye (BODIPY493) confirmed it (**Fig 1I, middle and right panel**).

We also used transmission electron microscopy (TEM) to observe the lipid droplet (LD) where the synthesized TG is packaged, which is acquired by VLDL precursor to form mature VLDL particles. The results showed that the numbers of LDs with larger ovoid structures were obviously increased, with the expanded volume, in the hepatocytic cytoplasm of the *Lzp*^*-/-*^ mice fed a HFD, where no aberrant mitochondria or ER morphology were observed (**Fig 1J and S2E and S2F Fig**).

These collective data indicated that LZP deficiency caused hepatic TG accumulation, as evidenced by TG mass quantitation, both oil red O and BODIPY493 staining, and TEM imaging.

### LZP may directly regulate TG and fatty acid metabolism

**Fig 2:**
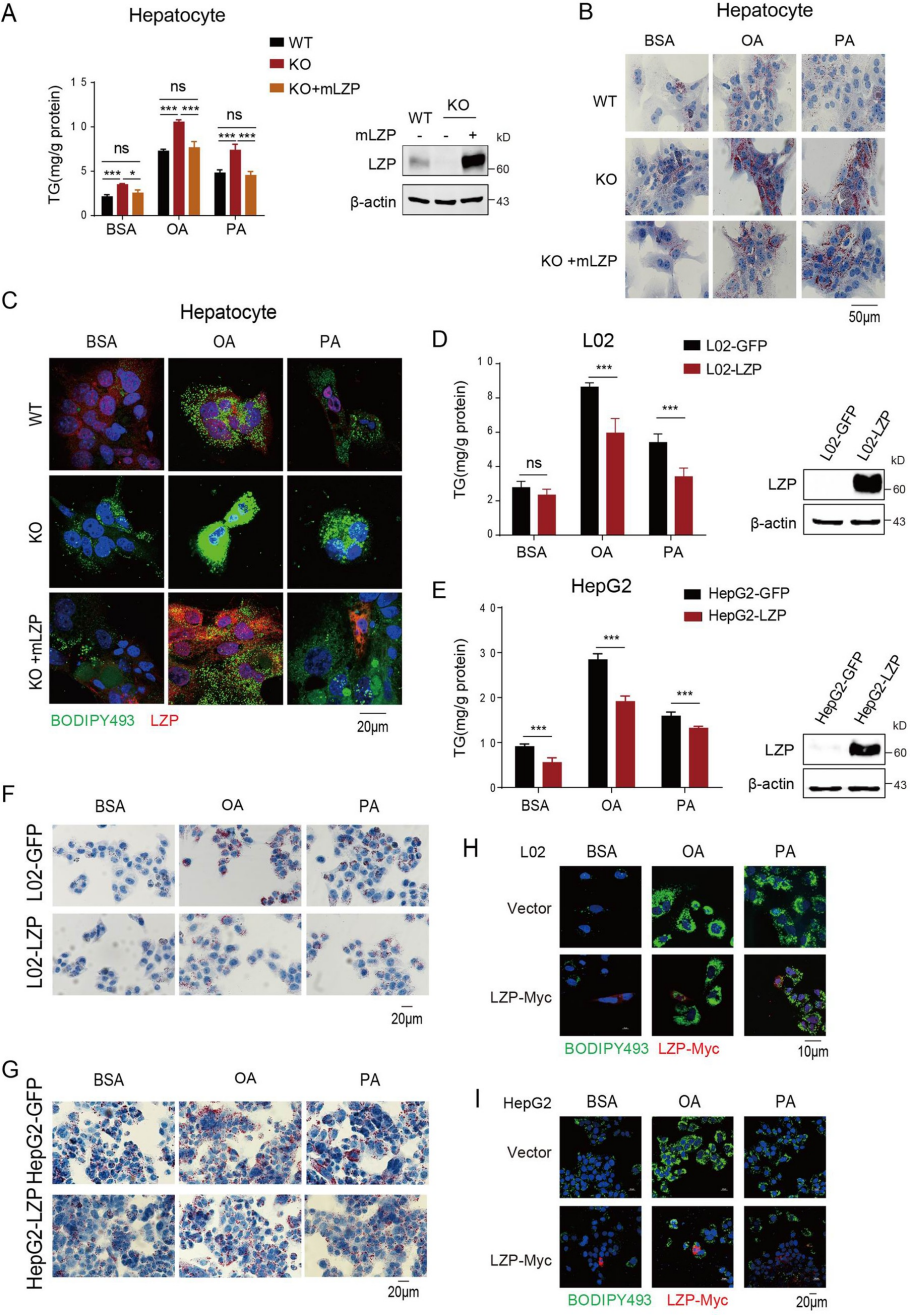
LZP deficiency causes hepatic TG accumulation. (A) TG contents of isolated hepatocytes was measured (left panel) and LZP expression were analyzed by Western blotting assay (right panel). Primary hepatocytes isolated from WT or *Lzp* KO mice were transfected with 3 μg plasmids containing mLZP-myc or empty vehicle for 24 h, and then followed 0.2 mM OA or PA treatments for 16 h. Finally, the cells were harvested for TG contents quantification and LZP expression analysis. (B) Images of Oil Red O (ORO) staining of hepatocytes isolated from WT and *Lzp* KO mice. The hepatocytes were transfected with 3 μg plasmids containing mLZP-myc or empty vehicle for 24 h, then followed 16 h treatments of 0.2 mM OA or PA to enhance lipid formation. Scale bar represents 50μm. (C) Images of immunofluorescence staining of LZP (red), LDs (stained with Bodipy 493, green), and nucleus (blue) of hepatocytes isolated from WT and *Lzp* KO mice. The hepatocytes were treated as above. Scale bar, 20μm. (D-E) Quantification of the cellular TG contents of L02 or HepG2 cells. Both cells stably expressed LZP or GFP were treated with 0.25 mM OA or PA for 16 h, and their ectopic expressions of LZP were validated by Western blot assay. (F-G) Oil Red O (ORO) staining of L02 cells or HepG2 cells, which stably expressed LZP or GFP were treated with 0.25mM OA or PA overnight. Scale bar, 20μm. (H, I) Immunofluorescence staining of LZP (red) and lipid droplets (green) of L02 or HepG2 cells, which were transfected with plasmids containing LZP-myc or empty vehicle, followed by 0.25mM OA or PA treatments overnight. The photos were taken by Nikon & A1Si laser scanning confocal microscope. Scale bar, 10 μm(H) or 20 μm(I). Data were expressed as means ± SEM, and statistically analyzed by student’s t-test, *P < 0.05, **P < 0.01. Numerical values for each of the experiments represented are available in **S4 Table**.

To exclude the effect of physical TG or other metabolites leading to increased TG in the livers, we isolated hepatocytes from 10-week-old *Lzp*^*-/-*^ and WT mice to examine lipid metabolism. As expected, the TG level was significantly increased in LZP-deficient hepatocytes compared to that in the WT hepatocytes. Remarkably, the rescue experiment showed that ectopic LZP expression significantly reduced the TG level (**Fig 2A and S4 Table**), an outcome consistent with the oil red O staining (**Fig 2B**) and BODIPY493 fluorescence dye-staining (**Fig 2C**) results. This finding suggested that LZP regulates hepatic TG levels in a physical environment-independent manner.

Furthermore, we treated these isolated mouse hepatocytes with oleic acid (OA), an unsaturated fatty acid, or palmitic acid (PA), a saturated free fatty acid, which simulates excessive influx of free fatty acids into cells. The results showed more TG accumulation in LZP-deficient hepatocytes than in WT hepatocytes, whereas ectopic LZP expression eliminated the accumulated TG in the hepatocytes, a finding supported by oil red O and BODIPY493 fluorescence dye staining (**Fig 2A–2C and S4 Table**).

Moreover, ectopic expression of LZP in the human fetal hepatic cell line L02 and hepatoma cell line HepG2, which have undetectable endogenous LZP, led to decreased TG levels under normal culture conditions, OA treatment, or PA treatment (**Fig 2D and 2E and S4 Table**), findings consistent with the oil red O and BODIPY493 staining **(Fig 2F–2I)**.

All these data revealed that LZP directly affects TG metabolism and that the presence of LZP can reduce TG accumulation within hepatocytes, possibly by promoting TG transportation from the cytoplasm to the extracellular space.

### LZP is required for TG secretion

To explore the mechanisms underlying the LZP deficiency induced hepatic TG increase, we examined the mRNA levels of the key genes encoding the proteins involved in hepatic *de novo* lipogenesis, triglyceride degradation, and lipoprotein-VLDL maturation and secretion in livers from *Lzp*^*-/-*^ mice. However, no significant differences were observed in gene expression in the *Lzp*^*-/-*^ and WT mice (**S3A–S3C Fig and S5 Table**), indicating that hepatic TG accumulation in the *Lzp*^*-/-*^ mice could not be ascribed to the dysregulated expression of these select genes.

**Fig 3:**
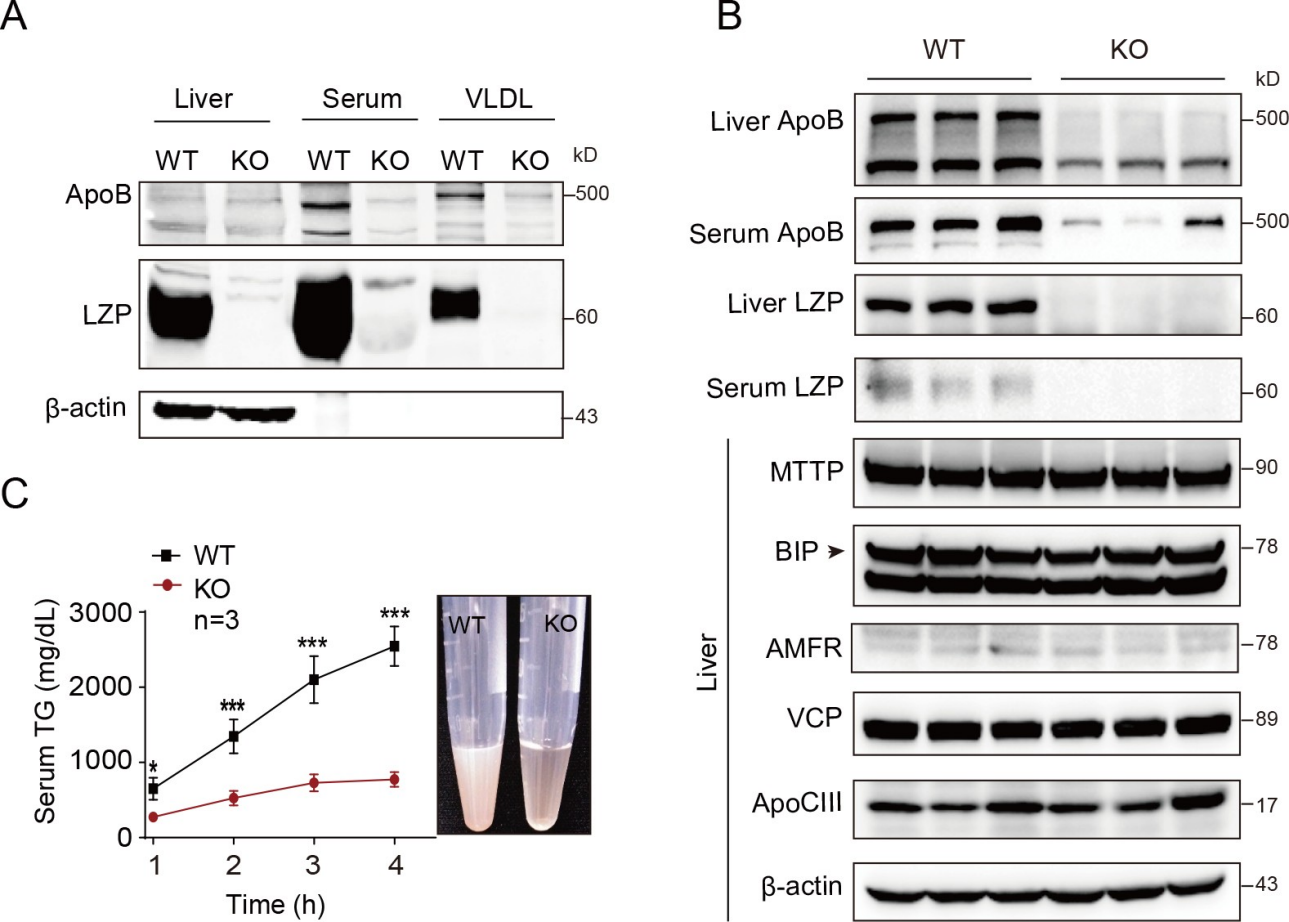
LZP is required for TG secretion. (A) Both LZP and apoB were measured by Western blot assay in livers, serum, and VLDL particles from WT and KO mice. VLDL were isolated via isopycnic KBr gradient method. (B) *Lzp* deficiency decreased apoB level in hepatocyte and serum. The key proteins involved in VLDL assembly, transportation, and secretion were analyzed with indicated antibodies (n = 3). (C) LZP deficiency decreased TG secretion. WT and *Lzp*^*-/-*^ mice were injected with 500 mg/kg Triton WR-1339 after 8 h fasting. Blood samples were collected at the indicated time points for TG detection (n = 3). Data were expressed as means ± SEM, and statistically analyzed by student’s t-test or one-way ANOVA, *P < 0.05, **P < 0.01. Numerical values for each of the experiments represented are available in **S6 Table**.

Next, we proposed that LZP may affect the TG transportation. To validate this hypotheses, we examined the key molecules known to be associated with TG transportation, namely, apoB, MTTP, BIP, AMFR, VCP and apoCIII, which are involved in VLDL assembly, transportation and secretion [17,18]. Interestingly, apoB100, a protein crucial for TG-rich VLDL assembly, transportation and secretion, was dramatically decreased in the livers, serum and isolated VLDL particles from the *Lzp*^*-/-*^ mice (**Fig 3A and 3B**). The apoB48, a known molecule expressed in rodent liver, slightly decreased in the liver of *Lzp*^*-/-*^ mice. However, in contrast to the change in apoB, the changes of other related proteins were not observed (**Fig 3B**).

Notably, similar to apoB, LZP was detected in serum, as well as VLDL particles (density < 1.006) isolated from the WT mice according to the method developed by Richard J [19] (**Fig 3A**), strongly suggesting that LZP involves in TG-rich VLDL assembly or secretion. To verify the proposed concept, we first monitored the rate of *in vivo* TG secretion in *Lzp*^*-/-*^ mice by intravenously injecting Triton WR-1339 to block VLDL catabolism [20]. Under this condition, the serum TG, which is mainly derived from hepatic VLDL secretion, was markedly and significantly decreased in the *Lzp*^*-/-*^ mice, by as much as approximately 70%, compared to that in the WT mice (**Fig 3C, left panel and S6 Table**). Consistently, the serum from the *Lzp*^*-/-*^ mice was obviously clearer than that taken from the WT mice (**Fig 3C, right panel**). The data described above indicate that LZP is required for hepatic TG-rich VLDL secretion.

### LZP directly interacts with ApoB

**Fig 4:**
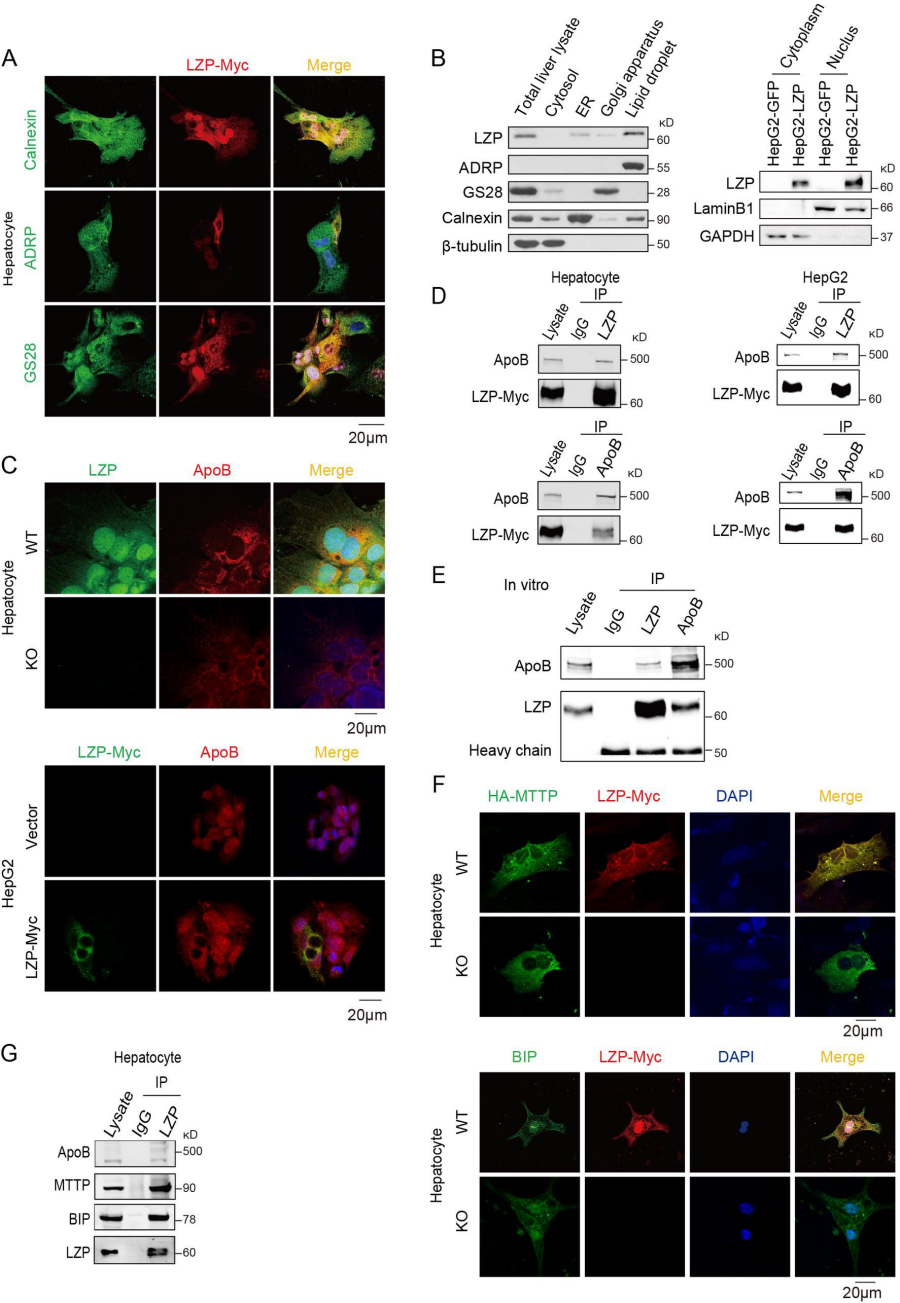
LZP interacts with apoB. (A) Co-localization of LZP with Calnexin (ER marker), ADRP (lipid marker), or GS28 (Golgi marker) in hepatocytes from WT mice. Scale bar, 20 μm. (B) LZP expression levels in cytoplasm, nucleus, and different organelle isolated from livers of WT mice. (C) Co-localization of LZP and apoB in hepatocytes from WT mice or in HepG2 cells overexpressed LZP. Scale bar, 20 μm. (D) Co-immunoprecipitation of LZP and apoB in hepatocytes or in human HepG2 cells overexpressed LZP. (E) *In vitro* co-immunoprecipitation of commercial apoB and purified LZP. (F) Co-localization of the ectopic LZP (red) and MTTP (green) (Upper panel) or endogenous BiP (green) (lower panel) in hepatocytes. Scale bar, 20 μm. (G) Co-immunoprecipitations of endogenous LZP and apoB, MTTP, or BIP in hepatocytes isolated from WT mice.

TG is synthesized predominantly on the ER where apoB is translated on ribosomes and then translocated across the membrane to the ER lumen [21]. ApoB recruits TG to form pre-VLDLs and transfers to the Golgi apparatus for maturation and subsequently secretion. In the present study, the fluorescence immunostaining assays showed that ectopic LZP was colocalized with Calnexin (an ER-specific marker), ADRP (a lipid droplet marker), and GS28 (a trans-Golgi specific marker) in the mouse hepatocytes (**Fig 4A**), implying that LZP can localize to these organelles, which are related to VLDL assembly and secretion. The Western blot assay of nucleus and different subcellular organelles confirmed that LZP existed in the nucleus, ER, LDs, and Golgi apparatus, being especially high in the LDs where synthesized TG is packaged (**Fig 4B, left panel**).

Subsequently, we examined the localizations of LZP and apoB, using immunofluorescence staining. The data showed that endogenous and ectopic LZP colocalized with apoB within the cytoplasm of the mouse hepatocytes and human HepG2 cells (**Fig 4C**). Furthermore, we observed that both ectopic LZP and apoB reciprocally immunoprecipitated each other in the hepatocytes and HepG2 cells (**Fig 4D**). Moreover, *in vitro* coimmunoprecipitation with purified LZP and apoB demonstrated that LZP directly interacted with apoB (**Fig 4E**).

In addition to that of LZP and apoB, the ectopic LZP also colocalized with and immunoprecipitated MTTP, one of the key proteins in the synthesis and secretion of apoB-containing lipoproteins, and BIP, an ER-resident luminal chaperone that engages apoB polypeptides [22,23], in mouse hepatocyte (**Fig 4F and 4G**).

These collective data indicated that LZP may regulate TG-rich VLDL assembly, transportation and secretion by directly interacting with apoB as well as its associated proteins, MTTP and BIP.

### LZP maintains ApoB stability

**Fig 5:**
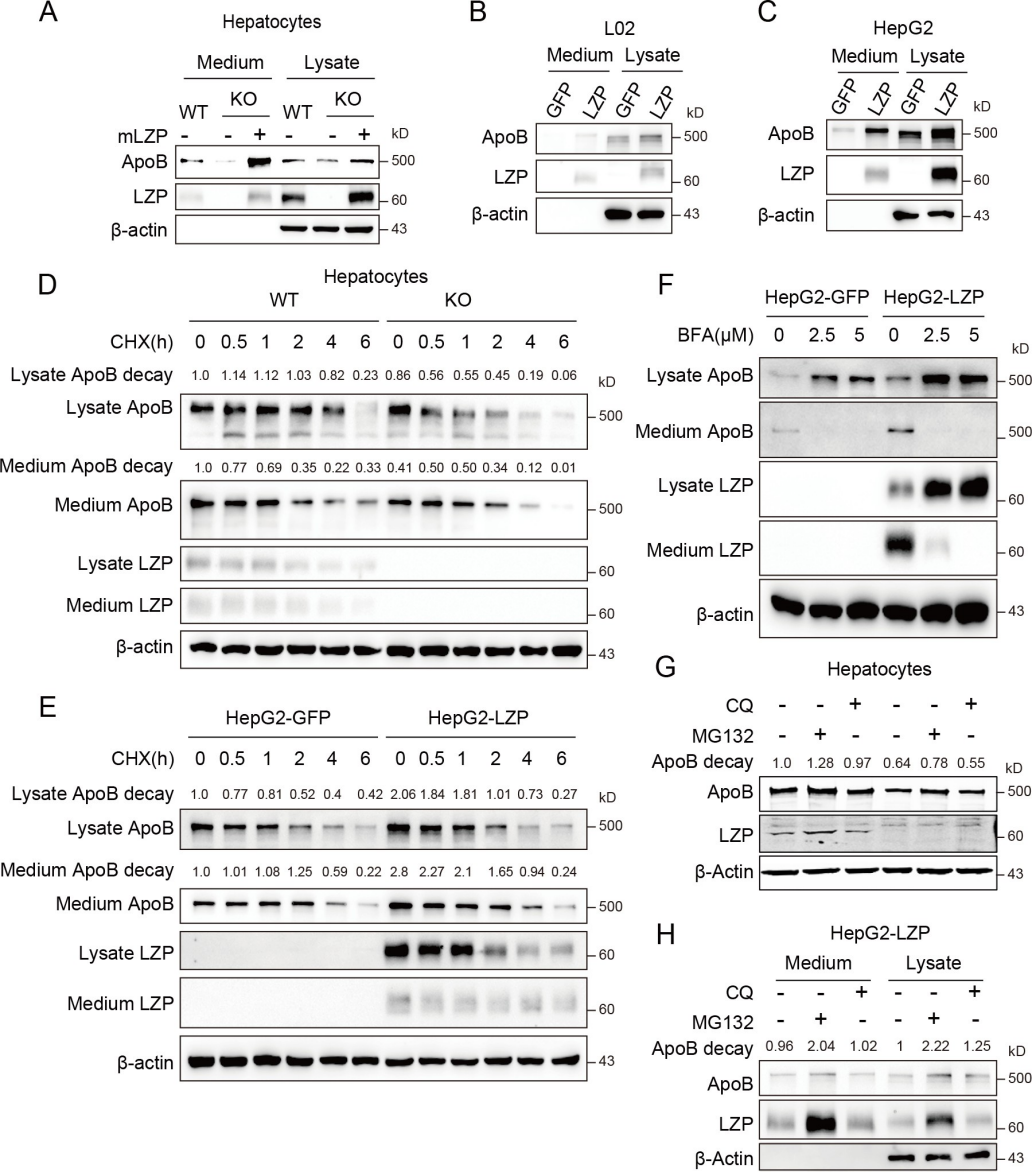
LZP maintains apoB stability. (A) LZP deficiency decreased the cellular and secreted apoB levels from hepatocytes, whilst LZP overexpression recovered apoB level. (B-C) Ectopic LZP expression increased cellular and secreted apoB levels from both L02 (B) and HepG2 cells (C). (D) LZP deficiency decreased the half-life of apoB in hepatocytes. Hepatocytes were treated with 100 μg/ml CHX for the indicated times. (E) Ectopic LZP extended half-life of apoB in HepG2 cells. HepG2 cells overexpressed GFP or LZP were treated with 100 μg/ml CHX for the indicated times. The apoB’s integral optical density of each time point was normalized by 0 time and value was labeled at the upper of band. (F) Blocking ER-Golgi trafficking with brefeldin A (BFA) reduced apoB and LZP in cell culture medium. (G) MG132, not CQ, blocked the degradation of apoB in hepatocytes. Hepatocytes were treated with 20μM MG132 or 50μM CQ for 24h. (H) MG132 blocked the degradation of cellular and secreted apoB as well as LZP from HepG2-LZP cells. Numerical values for each of the experiments represented are available in **S8 Table**.

During VLDL assembly and secretion, the polypeptide quality and quantity of apoB are regulated by certain proteins, such as MTTP, BIP, AMFR and Cideb [22–25]. The results described above suggested that LZP might regulate apoB stability. To validate this supposition, we forced the ectopic expression of LZP in hepatocytes isolated from 10-week-old *Lzp*^-/-^ mice. Interestingly, the ectopic LZP expression recovered the apoB levels of both cellular and secreted fractions, indicating that LZP stables apoB, subsequently increases its secretion **(Fig 5A)**. Moreover, ectopic LZP expression obviously increased both cellular and secreted apoB fractions of human L02 and HepG2 cells **(Fig 5B and 5C)**. However, the mRNA level of apoB in the livers and hepatocytes from the *Lzp*^*-/-*^ mice was not significantly changed compared to level in the WT mice (**S3C and S4A Figs and S5 and S7 Tables**), and ectopic LZP expression did not promote the transcription of apoB in the HepG2 cells (**S4B Fig and S7 Table**). These data indicate that LZP may affect apoB stability, not its secretion, possibly through translation or posttranslational modification processes, but not transcription.

To confirm the effect of LZP on apoB stability, we examined the dynamic change of intracellular and secreted apoB from WT and *Lzp*^*-/-*^ hepatocytes, which were treated with the translation inhibitor cycloheximide (CHX). The data showed that the intracellular and secreted apoB levels from *Lzp*^*-/-*^ hepatocytes were obviously decreased compared with WT hepatocytes at all examined time points (**Fig 5D and S8 Table**). Further, we ectopically expressed LZP and examined the dynamic change of apoB in HepG2 cells. The data showed that the intracellular and secreted apoB levels were also increased at all examined time points after ectopic LZP expression in HepG2 cells (**Fig 5E and S8 Table**). The above data suggest that LZP deficiency decreased the half-life of apoB via posttranslational modification. However, the overexpression of LZP may reverse it **(Fig 5D and 5E and S8 Table)**.

Furthermore, we added brefeldin A (BFA) to block ER-Golgi trafficking and then examined apoB and LZP in cell lysate and medium. The data showed that both apoB and LZP of medium were reduced after BFA treatment **(Fig 5F and S8 Table)**. In addition, we used the ubiquitin-proteasome inhibitor MG132 and the lysosome inhibitor chloroquine diphosphate salt (CQ) to determine whether either contributes to the observed LZP deficiency-induced apoB degradation. Interestingly, only MG132, but not CQ, restored the apoB level of the hepatocytes from *Lzp*^*-/-*^ mice **(Fig 5G and S8 Table**), and MG132 (not CQ) increased both intracellular and secreted LZP and apoB fractions of HepG2 cells with stably expressed ectopic LZP **(Fig 5H and S8 Table).** These data revealed that LZP could regulate apoB stability via the ubiquitin-proteasome degradation pathway.

### LZP attenuates AMFR-dependent ApoB degradation

**Fig 6:**
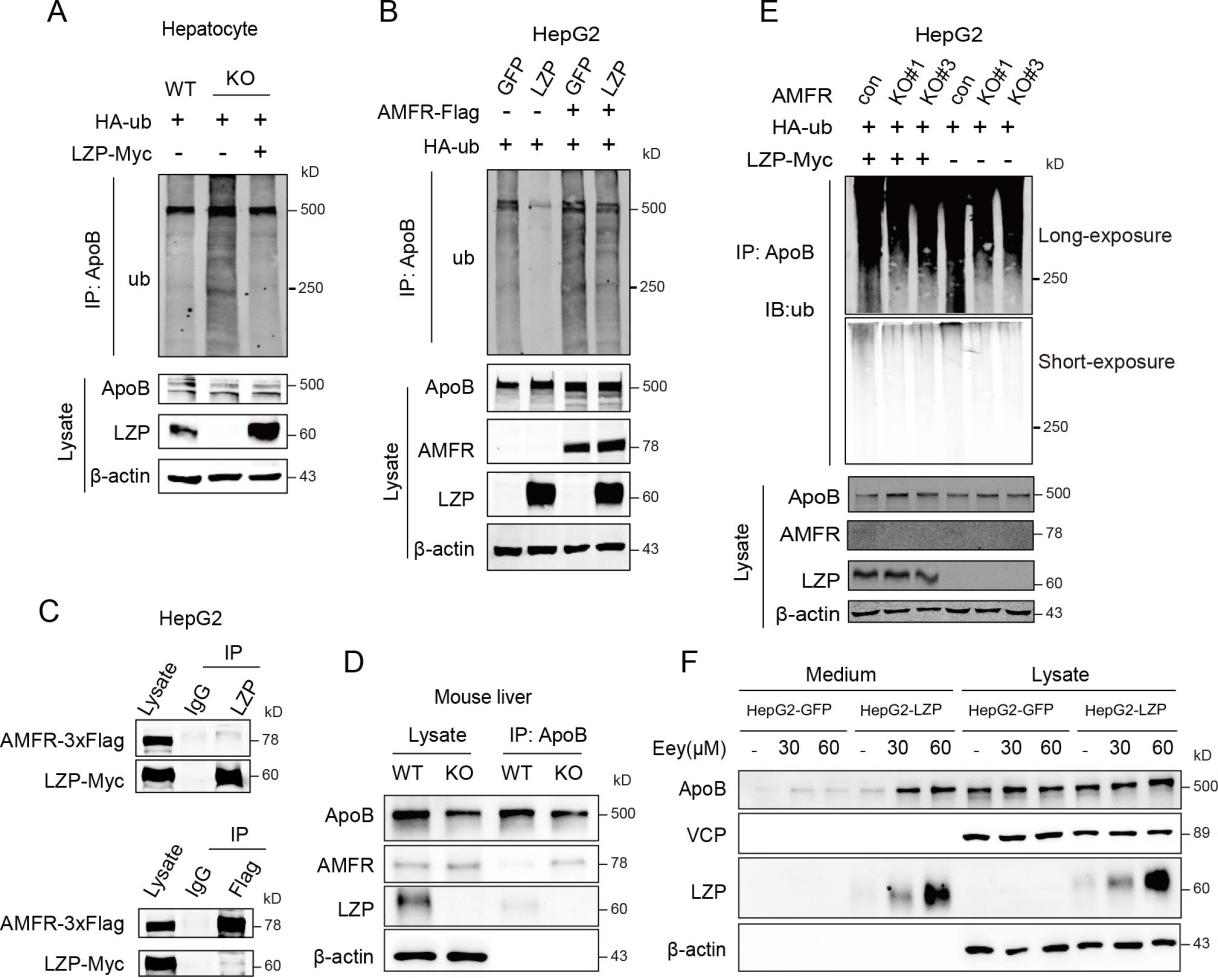
LZP attenuates AMFR-dependent apoB degradation. (A) LZP deficiency increased ubiquitination of apoB in hepatocytes, while ectopic LZP overexpression decreased it. Hepatocyte from WT or *Lzp* KO mice were transfected with mLZP-myc and HA-ub for 24 h, then treated with 20 μM MG132 for another 6 h. (B) Ectopic LZP inhibited apoB ubiquitination in HepG2 cells, while the AMFR overexpression increased apoB ubiquitination. (C) Co-immunoprecipitation of LZP and AMFR with indicated antibodies in HepG2 cells ectopically expressed AMFR and LZP. (D) LZP deficiency enhanced the apoB-AMFR interaction in liver. (E) Overexpressed LZP did not decrease the apoB ubiquitination level in the absence of AMFR in HepG2 cells. (F) ERAD inhibitor Eeyarestatin I (Eey) increased the intracellular and secreted apoB levels in both HepG2-GFP and HepG2-LZP cells. The cells were treated with Eey for 24h.

To further assess the effect of LZP on apoB stability, we first evaluated the apoB ubiquitination change in *Lzp*^*-/-*^ hepatocytes, and found that LZP loss caused an increase of apoB ubiquitination, which could be reversed by ectopic LZP **(Fig 6A)**. Consistently, ectopic LZP also inhibited apoB ubiquitination in the human HepG2 cells **(Fig 6B)**. These data confirmed that LZP maintains apoB stability by regulating its ubiquitination.

It is known that misfolded or unnecessary apoB is ubiquitinated by the key E3 ligase AMFR, and then, apoB undergoes ubiquitin-proteasome degradation in the ERAD pathway [26,27]. In the present study, the findings from an immunofluorescence assay supported that the AMFR level was negatively associated with the apoB level in the mouse hepatocytes and HepG2 cells **(S5A Fig**) and that AMFR was colocalized with LZP in the mouse hepatocytes and HepG2 cells (**S5B Fig)**. Both LZP and AMFR reciprocally immunoprecipitated each other **(Fig 6C)**. Interestingly, the AMFR immunoprecipitated by apoB was increased in *Lzp*^*-/-*^ hepatocytes, while the apoB level was decreased (**Fig 6D**), indicating that LZP attenuated the interaction between AMFR and apoB.

The above data suggest that LZP regulates apoB level through the AMFR-mediated ERAD pathway. To evaluate it, we knocked out *AMFR* in the HepG2 cells **(S5C Fig)**. As expected, apoB ubiquitination was obviously decreased in the absence of AMFR, but the absence or presence of LZP did not affect the ubiquitination of apoB anymore **(Fig 6E**). Subsequently, we used Eeyarestatin I (Eey), an inhibitor of the ERAD pathway, to block the enzymatic activity of VCP and thus suppress the ubiquitin-induced degradation of apoB [28] in HepG2 cells. The data showed that Eey elevated the intracellular and secreted fractions of apoB in both HepG2-GFP and HepG2-LZP cells, which is consistent with the effect of LZP on apoB ubiquitination in the absence of AMFR **(Fig 6F)**.

These collective data indicated that LZP maintains apoB stability by preventing the AMFR-dependent ubiquitin-proteasome degradation, which leads to the transportation and secretion of TG-rich VLDL into blood.

### LZP is highly expressed in the livers of obese mice and human patients

**Fig 7:**
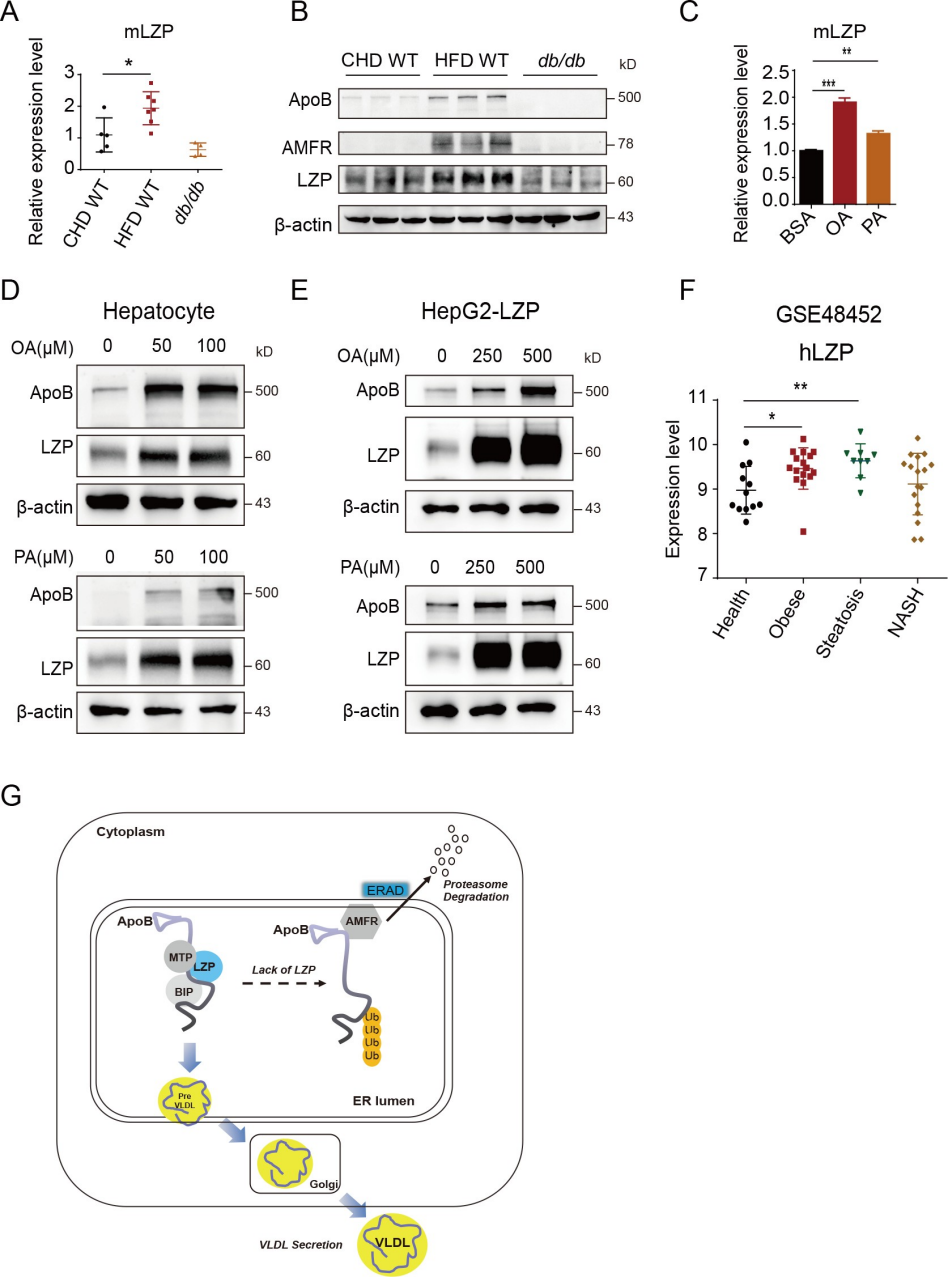
LZP highly expresses at livers of obese mice and patients. (A-B) *Lzp* mRNAs (A) and protein levels (B) in livers from mice fed with CHD or HFD, as well as *db/db* mice. (C-D) Both OA and PA increased hepatic *Lzp* mRNA (C) and protein levels (D). The hepatocytes separated from 10 weeks old mice were treated with OA or PA for 16 h, and then were harvested for real-time PCR and Western blot assays. (E) Both OA and PA increased LZP in HepG2 cells with ectopic LZP. (F) The analysis of *Lzp* mRNA levels in livers of obese, steatosis, and NASH patients using the dataset (GSE48452) excluded surgery patients. (G) Schematic illustration of the role of LZP in hepatic lipid metabolism. LZP interacts with apoB to attenuate its ubiquitination mediated by AMFP and subsequent proteasome degradation and to assistant VLDL assembly and secretion. Data were expressed as means ± SEM, and statistically analyzed by one-way ANOVA, *P < 0.05, **P < 0.01. Numerical values for each of the experiments represented are available in **S9 Table**.

The data described above demonstrated that LZP regulated hepatic lipid homeostasis and TG secretion into the blood by maintaining apoB stability. Therefore, LZP can be considered to be a critical factor against excess lipids formation in livers, such as is found in NAFLD that is accompanied by obesity or other metabolic syndromes. To validate whether LZP can serve as a response factor to lipid burden, we first analyzed LZP expression in the livers of different mouse models and observed that both mRNA and protein levels of LZP were significantly elevated in the mice fed a HFD but were decreased in diabetic *db/db* mice lacking functional leptin receptors, compared to the level in mice fed a CHD. Both apoB and AMFR were also increased in the mice fed a HFD but decreased in *db/db* mice **(Fig 7A and 7B and S9 Table)**. We then detected *Lzp* mRNA and protein levels in mouse hepatocytes treated with fatty acids OA and PA, and the data showed that the *Lzp* mRNA and protein levels were significantly increased, as was apoB (**Fig 7C and 7D**). Similarly, both OA and PA had obviously increased ectopic LZP expression under the CMV promoter in a dose-dependent manner in the human HepG2 cells, indicating that fatty acids promoted LZP expression not only at the transcriptional level but also at the translational level **(Fig 7E)**.

Consistently, the analysis of publicly accessible Gene Expression Omnibus (GEO) data sets excluded surgery patients demonstrated that the mRNA levels of *Lzp* in livers from obese and simple hepatic steatosis patients, but not nonalcoholic steatohepatitis (NASH), were also significantly higher than those of healthy controls **(Fig 7F and S9 Table)**.

The data support the notion that LZP might be a response factor against higher hepatic fatty acid and TG accumulation by preventing AMFR-mediated apoB ubiquitination and degradation, which leads to the transportation and secretion of excessive TG into blood to maintain hepatic lipid homeostasis **(Fig 7G)**.

## Discussion

Metabolic diseases such as obesity, insulin resistance, atherosclerosis, and NAFLD are often characterized by increased TG and cholesterol levels [12]. Dysregulation of hepatic lipid metabolism, including TG synthesis, storage, assembly, transportation and delivery via VLDL to other tissues, could contribute to the development of these diseases. ApoB is necessary for the assembly and secretion of TG-rich VLDL, which represents the rate-limiting steps in the production of hepatic VLDL. Interestingly, our study established a critical role for LZP in the regulation of hepatic TG metabolism by serving as a novel partner and regulator of apoB. Under normal conditions, LZP may attenuate the interaction between apoB and the ER-associated E3 ligase AMFR, preventing the AMFR-mediated ubiquitin-proteasome degradation of apoB. However, LZP deficiency enhances AMFR-mediated apoB ubiquitination and degradation, resulting in lower hepatic apoB levels, hepatic TG accumulation and reduced TG-rich VLDL secretion into the blood **(Fig 7G)**.

LZP belongs to the ZP-domain protein family, the members of which are found in various organs and tissues and play a wide variety of roles, from structural components to receptors to tumor suppressors [4,5]. LZP is a liver-specific ZP-domain protein that is secreted [7,9,10]. The findings from our previous study indicated that LZP is detectable in livers from embryonic to adult mice, implying that LZP plays a key role in the liver [7]. However, the function of LZP in the liver has not yet been discovered. Significantly, our current study reveals that LZP is required for hepatic TG-rich VLDL secretion, which represents a novel function of the ZP-domain protein family.

Many molecules have been found to be involved in lipid metabolism, such as GCKR for *de novo* lipogenesis; apoB, TM6SF2 and MTTP for VLDL assembly and secretion; PNPLA3(I148M) accumulation on lipid droplets; and the nuclear envelope-localized torsinA-LAP1 complex for hepatic VLDL secretion and steatosis [29–33]. Recently, Cideb, an ER and cytosolic lipid droplet (CLD)-associated protein, has been found to be involved in TG mobilization for VLDL assembly by interacting with apoB [25]. Among these known molecules, apoB is a key component for VLDL assembly because it provides the structural framework for the synthesis of TG-rich lipoproteins [19,21,34–36]; however, the detailed mechanisms and components have not all been determined. Interestingly, we discovered that LZP functions as a regulator of apoB, which participates in hepatic TG-rich VLDL secretion. Similar to the phenotype of Cideb-null mice, the LZP-deficient mice exhibit higher TG accumulation in their livers and lower serum TG levels. We verified that LZP attenuates the interaction of the E3 ligase AMFR and apoB and thereby prevents AMFR-mediated apoB ubiquitination and subsequent ubiquitin-proteasome degradation. The LZP protein was dramatically induced in the mouse livers by HFD feeding, whilst its transcription was only mildly induced (**Fig 7A and 7B and S9 Table**). In addition, Eey treatment strongly increased LZP protein level (**Fig 6F**). These data suggest that LZP itself may be degraded by ERAD. Given the interaction between LZP and AMFR (**Fig 6C**), LZP could be a substrate of AMFR as well.

LZP deficiency leads to hepatic TG accumulation in mice, as supported by the mass determination and histological morphology as evidences, including these observations through histochemical staining and electronic microscopy, indicating that LZP-deficient mice may develop a nonalcoholic fatty liver (NAFL). NAFLD encompasses a spectrum of diseases ranging from simple hepatic steatosis or NAFL, progressing through NASH and fibrosis, to cirrhosis and end-stage liver disease. According to the current paradigm, NAFL is considered to take a benign course, whereas chronic NAFL has the potential to progress to NASH. In our current study, no filtrated inflammatory cells were observed in the livers in the *Lzp*^*-/-*^ mice fed with either a CHD or HFD (up to 10 weeks). However, the role of LZP deficiency in NASH or more severe liver diseases, such as hepatocellular carcinoma (HCC), is worthy of further investigation because LZP is generally downregulated in HCC specimens.

Interestingly, HFD-fed mice showed the elevated LZP level in livers, but the diabetic mice (*db/db*) showed the decreased LZP in livers, indicating that the hepatocytes use different strategies or mechanisms under different conditions to regulate LZP expression. In our previous studies [6,7], we found that LZP highly expressed in livers and is almost undetectable at other tissues. This study suggests that the LZP deficiency caused hepatic lipid secretion disorder accompanied by low serum TG, the reduced adipose tissue, as well as less body weight at *Lzp*^-/-^ mice. However, the synthesized LZP in liver can be secreted to blood and subsequently be delivered to other tissues, and play a physiological role, such as a small amount of LZP detectable at kidney to regulate urate hemostasis [9,10]. It is possible that the secreted LZP could regulate adipose tissue mass, which might be confirmed in the future study. In general, our findings demonstrate that LZP, as a liver-specific ZP-domain protein, is required for TG-rich VLDL secretion because it protects apoB against AMFR-mediated ubiquitination and subsequent ubiquitin-proteasome degradation, profoundly influencing lipid homeostasis. However, LZP deficiency attenuates apoB-mediated VLDL secretion, leading to lower serum apoB and TG levels in mice; therefore, LZP could be considered a potential therapeutic target for hyperlipemia, especially hypertriglyceridemia.

## Materials and methods

### Ethics statement

All mouse experiments were approved by the Animal Care and Use Committee of Shanghai Jiao Tong University (Approval number: A2016008) and performed in accordance with the institutional guidelines and protocols.

### Mice and diets

*Lzp*^*-/-*^ (KO) mice, which were backcrossed with C57BL6/J (WT) over ten generations, were generated and maintained as previously described [10]. Six-week-old male *Lzp*^*-/-*^ and WT mice were given free access to a HFD (composed of 59% fat, 15% protein, and 26% carbohydrates based on caloric content) or a chow diet (composed of 10% fat, 20% protein, and 70% carbohydrates) for another 10 weeks.

### Blood and lipid parameters

The hepatic enzymes, including alanine amino transferase (ALT), aspartate amino transferase (AST), and alkaline phosphatase (ALP), and serum lipids were analyzed using a spectrophotometer (Chemix 180i). The triglyceride (TG) and cholesterol (TC) contents in liver and serum were measured following the manufacturer’s guidelines (290–63701 for TG, 294–65801 for TC, Wako).

### VLDL-TG and apoB secretion *in vivo*

After 8 h fasting, mice were injected TritonWR-1339 (500 mg/kg in PBS) through tail veins [20]. Fifty microliters of blood were drawn from the retro-orbital plexus into heparinized tubes at 1h intervals, and serum TG concentrations were measured.

### Ubiquitination analysis

HepG2 cell or primary hepatocytes were incubated with 20 μM MG132 for 6 h. Then, followed the coimmunoprecipitation assay to precipitate apoB protein with anti-apoB antibody and protein G agarose. The precipitated apoB was resolved in SDS-PAGE (5%), followed Western blotting analysis with anti-ubiquitin antibody.

### Quantitative PCR

RNAs were extracted from liver or hepatocytes with Trizol (Invitrogen) and were subjected to reverse transcription (Takara). The mRNA levels of genes involved in lipid *de novo* synthesis, oxidation, and secretion were detected by real-time PCR system (ABI). Primers used were listed in **S10 Table**. The relative expression levels of the respective genes were calculated using GAPDH as an internal control.

### Data analyses

All statistical analyses were performed with Graphpad prism software (version 6.01). Group differences were evaluated using two-tailed Student’s *t*-test or one-way analysis of variance (ANOVA). Different cut-off values, p < 0.05 (*), p < 0.01(**) and p < 0.001 (***), were considered significant.

Additional materials and methods were described in the **S1 Text and S11 Table.** Further information and requests for resources and reagents should be directed to and will be fulfilled by the Lead Contactor Ze-Guang Han (hanzg@sjtu.edu.cn).

## Supporting information

S1 Fig LZP-deficient mice exhibit lower serum triacylglycerol. Serum taken from 16 weeks old wild-type (WT, n = 5) or *Lzp* knock out (KO, n = 5) mice were harvest for biochemistry index test. Data shown are means ± SEM (student’s t-test). *P < 0.05, **P < 0.01. Numerical values for each of the experiments represented are available in **S1 Table**.
(TIF)

S2 Fig LZP deficiency causes hepatic TG accumulation. (A) The food intake of WT and *Lzp* KO mice fed CHD or HFD (n = 7). (B) TCHO levels of serum and liver were measured with wako TCHO test kit (n = 5–13). (C) Glucose tolerance test (GTT) and insulin tolerance test (ITT) of WT and KO mice fed CHD or HFD (n = 6–7). (D) Insulin receptor and its downstream signaling were analyzed with Western blot assays. Data shown are means ± SEM (student’s t-test or one-way ANOVA). *P < 0.05, **P < 0.01. (E, F) Transmission electron micrographs of liver sections from WT and *Lzp* KO mice fed with CHD or HFD. Scale bars represent 5μm or 500 nm. Numerical values for each of the experiments represented are available in **S2 Table**.
(TIF)

S3 Fig Examination of the key genes in liver lipid metabolism in LZP deficiency mice. (A-C) The mRNA analyses of key genes involved in hepatic lipid synthesis, oxidation, lipoprotein-VLDL maturation and secretion by real-time PCR assays (n = 3–7). Data were expressed as means ± SEM (student’s t-test). *P < 0.05, **P < 0.01. Numerical values for each of the experiments represented are available in **S5 Table**.
(TIF)

S4 Fig LZP maintains apoB stability. (A) The apoB mRNAs of primary hepatocytes from WT and *Lzp* KO mice were analyzed by real-time PCR. (B) The mRNAs of apoB in HepG2 ectopically expressed LZP were analyzed by real-time PCR. Data were expressed as means ± SEM (student’s t-test). Numerical values for each of the experiments represented are available in **S7 Table**.
(TIF)

S5 Fig LZP attenuates AMFR-dependent apoB degradation. (A) Double immunostainings with AMFR and apoB antibodies in primary hepatocytes from WT mice and HepG2 cells overexpressed AMFR-Flag. Scale bar, 20μm. (B) Co-localization of LZP (green) and AMFR (red) in hepatocytes from mice (upper) and HepG2 cells (below). Scale bar, 20μm. (C) Western blot checked the AMFR in AMFR knock out HepG2 cells by Crispr/Cas9.
(TIF)

S1 Table Numerical data of experiments shown in S1 Fig. Tables including numerical values of the experiments represented in **S1 Fig**.
(XLSX)

S2 Table Numerical data of experiments shown in S2 Fig. Tables including numerical values of the experiments represented in **S2 Fig**.
(XLSX)

S3 Table Numerical data of experiments shown in Fig 1. Tables including numerical values of the experiments represented in **Fig 1**.
(XLSX)

S4 Table Numerical data of experiments shown in Fig 2. Tables including numerical values of the experiments represented in **Fig 2**.
(XLSX)

S5 Table Numerical data of experiments shown in S3 Fig. Tables including numerical values of the experiments represented in **S3 Fig**.
(XLSX)

S6 Table Numerical data of experiments shown in Fig 3. Tables including numerical values of the experiments represented in **Fig 3**.
(XLSX)

S7 Table Numerical data of experiments shown in S4 Fig. Tables including numerical values of the experiments represented in **S4 Fig**.
(XLSX)

S8 Table Numerical data of experiments shown in Fig 5. Tables including numerical values of the experiments represented in **Fig 5**.
(XLSX)

S9 Table Numerical data of experiments shown in Fig 7. Tables including numerical values of the experiments represented in **Fig 7**.
(XLSX)

S10 Table Primers used in the present manuscript. Tables including primers used in the present manuscript.
(XLSX)

S11 Table Key Resources used in the present manuscript. Collected information of antibodies, reagents and assay kits used in the present manuscript.
(XLSX)

S1 Text Supplementary materials and methods. (DOCX)
