# Supplementary figures and images for: LZP is required for hepatic triacylglycerol transportation through maintaining apolipoprotein B stability
Source: PLoS Genet. 2021 Feb 16;17(2):e1009357. doi: 10.1371/journal.pgen.1009357 (PMC7909667; doi:10.1371/journal.pgen.1009357)

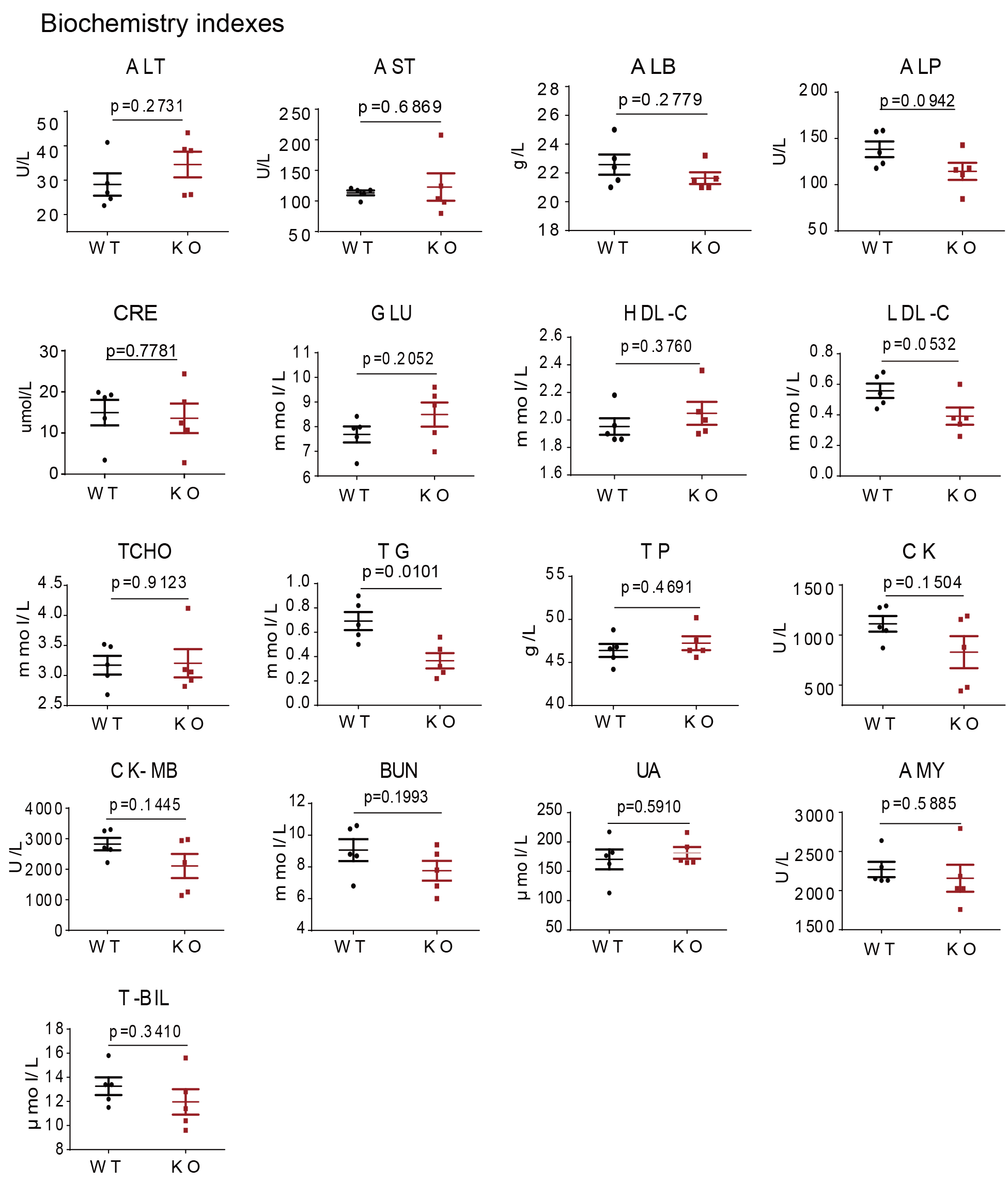

Supplement: S1 Fig — Serum taken from 16 weeks old wild-type (WT, n = 5) or Lzp knock out (KO, n = 5) mice were harvest for biochemistry index test. Data shown are means ± SEM (student’s t-test). *P < 0.05, **P < 0.01. Numerical values for each of the experiments represented are available in S1 Table. (TIF) [file pgen.1009357.s001.tif]

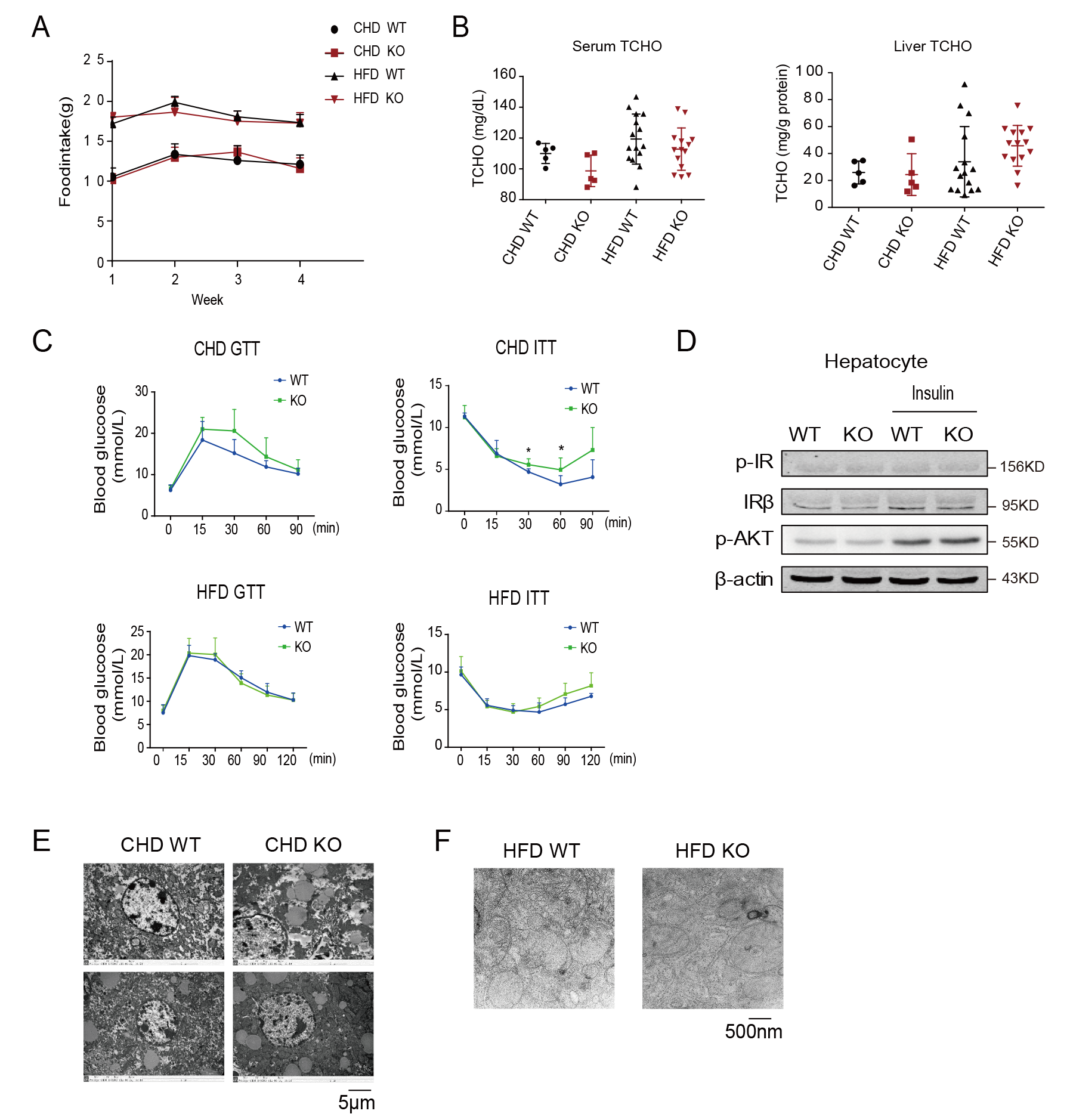

Supplement: S2 Fig — (A) The food intake of WT and Lzp KO mice fed CHD or HFD (n = 7). (B) TCHO levels of serum and liver were measured with wako TCHO test kit (n = 5–13). (C) Glucose tolerance test (GTT) and insulin tolerance test (ITT) of WT and KO mice fed CHD or HFD (n = 6–7). (D) Insulin receptor and its downstream signaling were analyzed with Western blot assays. Data shown are means ± SEM (student’s t-test or one-way ANOVA). *P < 0.05, **P < 0.01. (E, F) Transmission electron micrographs of liver sections from WT and Lzp KO mice fed with CHD or HFD. Scale bars represent 5μm or 500 nm. Numerical values for each of the experiments represented are available in S2 Table. (TIF) [file pgen.1009357.s002.tif]

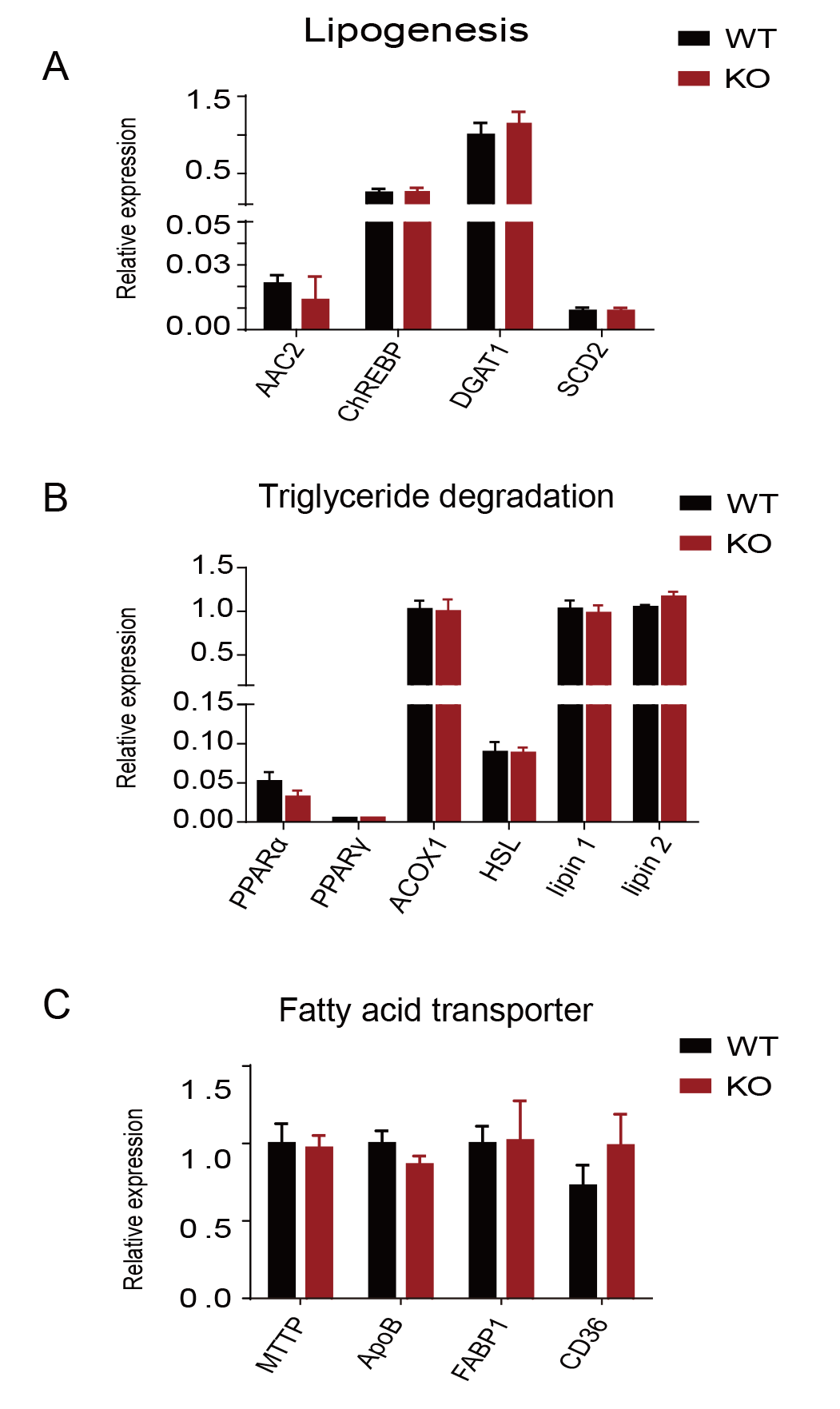

Supplement: S3 Fig — (A-C) The mRNA analyses of key genes involved in hepatic lipid synthesis, oxidation, lipoprotein-VLDL maturation and secretion by real-time PCR assays (n = 3–7). Data were expressed as means ± SEM (student’s t-test). *P < 0.05, **P < 0.01. Numerical values for each of the experiments represented are available in S5 Table. (TIF) [file pgen.1009357.s003.tif]

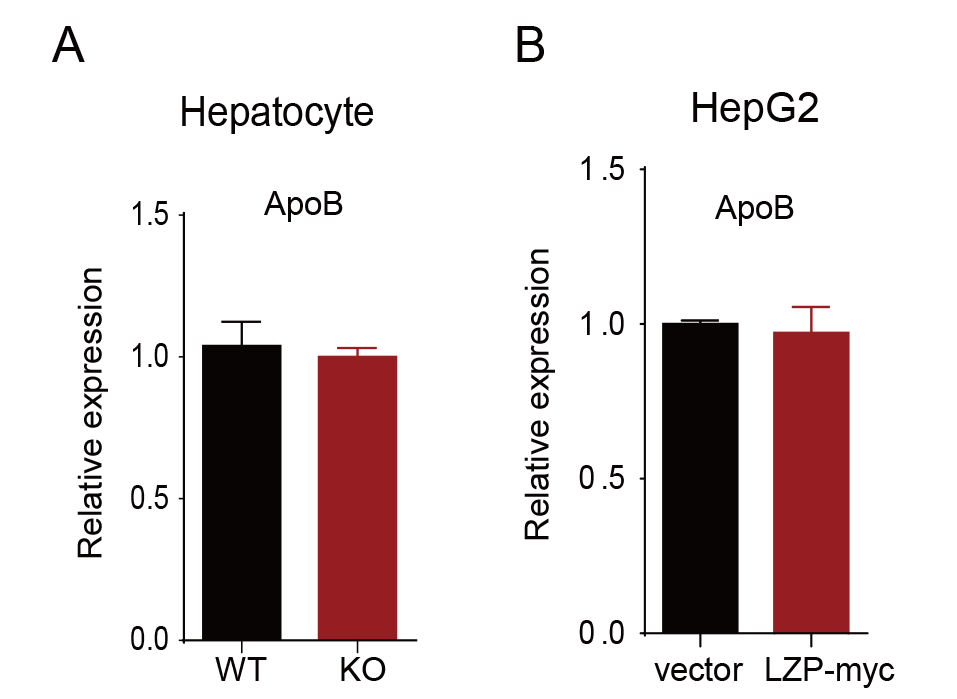

Supplement: S4 Fig — (A) The apoB mRNAs of primary hepatocytes from WT and Lzp KO mice were analyzed by real-time PCR. (B) The mRNAs of apoB in HepG2 ectopically expressed LZP were analyzed by real-time PCR. Data were expressed as means ± SEM (student’s t-test). Numerical values for each of the experiments represented are available in S7 Table. (TIF) [file pgen.1009357.s004.tif]

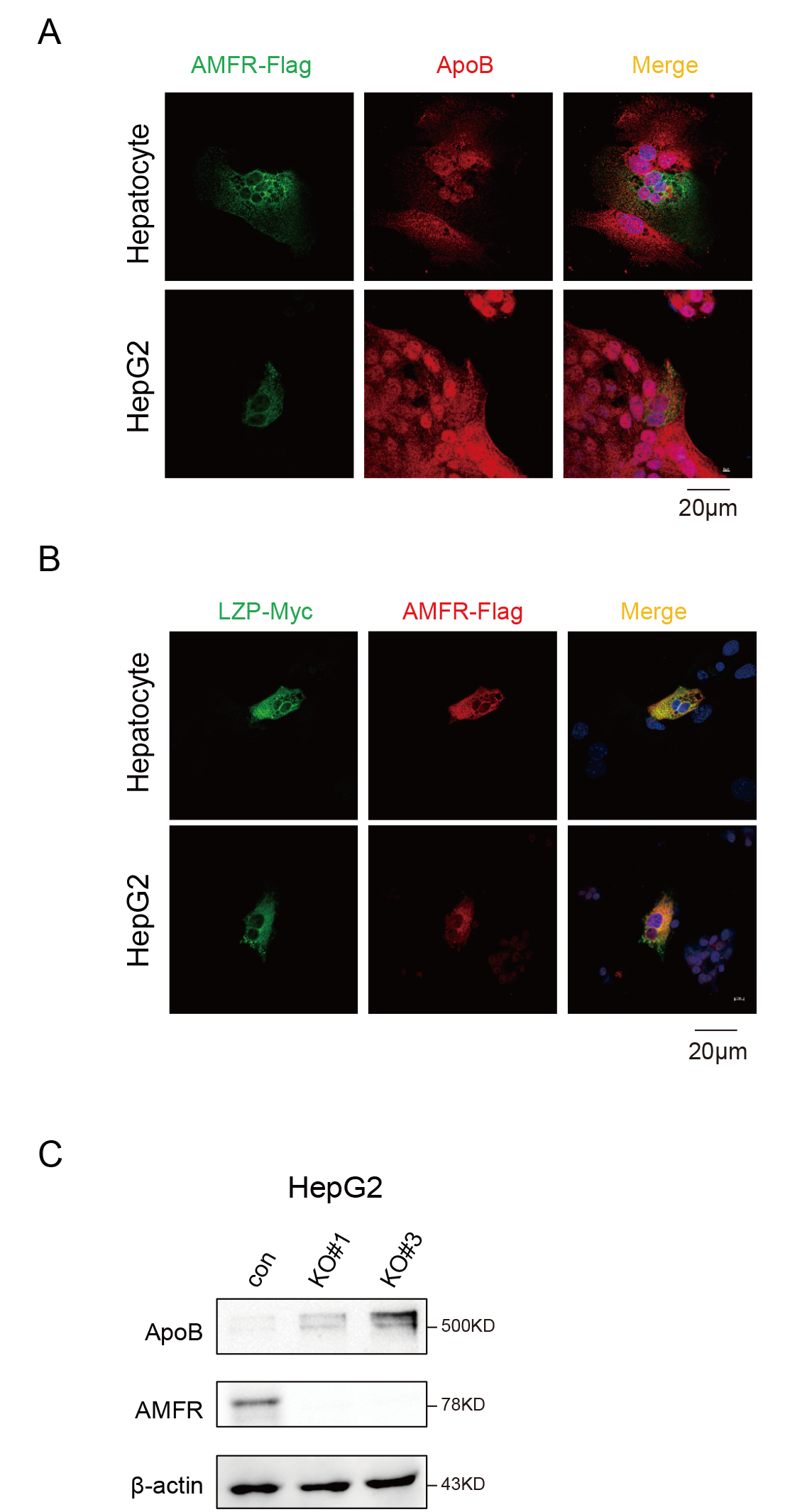

Supplement: S5 Fig — (A) Double immunostainings with AMFR and apoB antibodies in primary hepatocytes from WT mice and HepG2 cells overexpressed AMFR-Flag. Scale bar, 20μm. (B) Co-localization of LZP (green) and AMFR (red) in hepatocytes from mice (upper) and HepG2 cells (below). Scale bar, 20μm. (C) Western blot checked the AMFR in AMFR knock out HepG2 cells by Crispr/Cas9. (TIF) [file pgen.1009357.s005.tif]
