# Supplementary material for: LZP is required for hepatic triacylglycerol transportation through maintaining apolipoprotein B stability
Source: PLoS Genet. 2021 Feb 16;17(2):e1009357. doi: 10.1371/journal.pgen.1009357 (PMC7909667; doi:10.1371/journal.pgen.1009357)
Supplement: S1 Text — (DOCX) [file pgen.1009357.s017.docx]

**Supplementary materials and methods**

1. **Cell culture and treatment**

The immortalized human fetal liver cell line L02 was obtained from Shanghai Chang-Zheng Hospital, the Second Military Medical University in China. HepG2 was purchased from ATCC. Primary hepatocytes were isolated from 10-week-old mice following the literature’s method [[1](#_ENREF_1)]. All cells were cultured in DMEM supplemented with 10% fetal bovine serum (FBS) and 100 units of penicillin-streptomycin, and maintained at 37°C with 5% CO2 atmosphere in a humidified incubator. To stimulate lipid accumulation *in vitro*, cells were treated with 0.2 mM palmitate acid or 0.2 mM oleic acid for 16 h.

1. **Plasmids and transfection**

The plasmids encoding hLZP-myc was constructed from the cDNA of human liver as described previously [[2](#_ENREF_2), [3](#_ENREF_3)]. The plasmid expressing Flag-tagged human AMFR is a gift from professor Bao-Liang Song, Wuhan University. The plasmid expressing HA-tagged human MTTP was constructed by cloning MTTP cDNA to pcDNA3.0 vector as described previously [[4](#_ENREF_4)]. Transient transfection assays were performed using Lipofectamine 2000 (Invitrogen) according to the manufacturer's protocol. Virus expression LZP was constructed by subcloning human LZP with Flag tag at 3’ end into pCDH-CMV-MCS-EF1-copGFP (CD511B-1) vector, and lentivirus was packaged at HEK293T cells following the manufacturer’s protocol. HepG2 or L02 cells were infected with virus, and then the positive single cells were selected with puromycin and amplified.

1. **Histological staining**

Liver sections were embedded in paraffin and stained with hematoxylin and eosin (H&E) to visualize hepatocytes, inflammatory cells, and cellular organelles (lipid droplets). To evaluate mouse liver lipid accumulation, frozen liver sections of 8~10μm were fixed in formalin, rinsed with 60% isopropanol, and then stained with freshly prepared oil red O solution (Sigma-Aldrich) or lipophilic fluorescent dye boron-dipyrromethene (BODIPY493). Oil red O stained sections were rinsed with 60% isopropanol and counterstained with hematoxylin. The images were taken by inverted microscope (Nikon A1Si).

1. **Immunoblotting**

The liver or cell’s proteins were separated by SDS-PAGE, transferred to nitrocellulose membranes. The specific proteins were visualized by immunoblotting with respective primary and secondary antibodies. The following antibodies were used in current study: ApoB (ab20737, abcam), ubiquitin (sc-9133, Santa Cruz), Calnexin (sc-70481, Santa Cruz), GS28 (sc133148, Santa Cruz), ADRP (sc-32450, Santa Cruz), β-tublin (sc-5274, Santa Cruz), BIP (WL03157, Wanleibio), PCSK9 (WL03068, Wanleibio), β-actin (A5441, Sigma). Antibodies from Abcam (ab139190) and produced in our lab as described previously [[5](#_ENREF_5)] were used to detect endogenous and ectopic LZP, respectively. All HRP conjugated secondary antibodies were purchased from Sigma, and fluoresces conjugated secondary antibodies were purchased from Life.

1. **Coimmunoprecipitation assay**

Cultured cells or liver tissues were lysated with IP specific lysis buffer (#9803，CST), indicated antibody and protein G-agarose beads were added to the cell lysates followed rocking at 4°C overnight. The beads were washed three times with lysis buffer, and resuspended in loading buffer, and boiled for 5 min. The protein samples were separated in SDS-PAGE gel, and transferred to an NC membrane (Millipore, USA) for Western blotting analysis with the specific antibodies.

1. **Immunofluorescence staining**

Briefly, cells were fixed in paraformaldehyde at 4℃ for 30 min, penetrated with 0.5% Triton X-100(Sigma-Aldrich) at room temperature for 5 min, and blocked with BSA(Sigma-Aldrich) for 30 min. Then the cells were incubated with primary antibodies for 2 h at room temperature, followed incubation of secondary antibodies conjugated with Alexa Fluor 488 or 647, respectively, for 1 h. Images were taken by Nikon A1Si laser scanning confocal microscope.

**References**

1. Kegel V, Deharde D, Pfeiffer E, Zeilinger K, Seehofer D, Damm G. Protocol for Isolation of Primary Human Hepatocytes and Corresponding Major Populations of Non-parenchymal Liver Cells. Journal of visualized experiments : JoVE. 2016(109):e53069.

2. Xu ZG, Du JJ, Cui SJ, Wang ZQ, Huo KK, Li YY, et al. Identification of LZP gene from Mus musculus and Rattus norvegicus coding for a novel liver-specific ZP domain-containing secretory protein. DNA Seq. 2004;15(2):81-7.

3. Xu ZG, Du JJ, Zhang X, Cheng ZH, Ma ZZ, Xiao HS, et al. A novel liver-specific zona pellucida domain containing protein that is expressed rarely in hepatocellular carcinoma. Hepatology. 2003;38(3):735-44.

4. Qu YL, Deng CH, Luo Q, Shang XY, Wu JX, Shi Y, et al. Arid1a regulates insulin sensitivity and lipid metabolism. EBioMedicine. 2019;42:481-93.

5. Shen HL, Xu ZG, Huang LY, Liu D, Lin DH, Cao JB, et al. Liver-specific ZP domain-containing protein (LZP) as a new partner of Tamm-Horsfall protein harbors on renal tubules. Mol Cell Biochem. 2009;321(1-2):73-83.
